# Inflammation and fibrosis at pancreatic resection margin and their role in post-operative pancreatic fistula development after pancreaticoduodenectomy: a pilot study from a single institution

**DOI:** 10.1016/j.sopen.2025.12.004

**Published:** 2026-01-02

**Authors:** Roberto Cammarata, Vincenzo La Vaccara, Alberto Catamerò, Chiara Taffon, Gianluca Costa, Laura Olivieri, Roberto Coppola, Damiano Caputo

**Affiliations:** a Operative Research Unit of General Surgery, Fondazione Policlinico Universitario Campus Bio-Medico, Rome, 00128, Italy; b Università Campus Bio-Medico di Roma, Italy; c Pathology Unit, Fondazione Policlinico Universitario Campus Bio-Medico, Via Alvaro del Portillo, 200, 00128, Roma, Italy; d Department of Life Sciences, Health and Health Professions, Link Campus University, Rome, 00165, Italy; e Research Unit of General Surgery, Università Campus Bio-Medico di Roma, Rome, 00128, Italy

**Keywords:** Pancreaticoduodenectomy (PD), Clinically relevant POPF (CR-POPF), Fibrosis, Chronic lymphomononuclear inflammatory infiltrate (CLII), Pancreatic resection margin (PRM), Frozen section

## Abstract

**Background/objectives:**

Postoperative pancreatic fistula (POPF) is a major complication after pancreaticoduodenectomy (PD), with significant impact on outcomes. While the absence of pancreatic fibrosis is a known risk factor, its intraoperative assessment is often subjective. Moreover, the potential protective role of chronic inflammation at the pancreatic resection margin (PRM) has not been fully explored. This study aimed to evaluate the histological presence of fibrosis and chronic lymphomononuclear inflammatory infiltrate (CLII) at the PRM as predictors of POPF and clinically relevant POPF (CR-POPF), and to assess their intraoperative feasibility via frozen sections.

**Materials and methods:**

A retrospective analysis was performed on 141 patients who underwent PD (2014–2022). Intraoperative frozen sections of the PRM were reviewed for fibrosis and CLII using standardized semi-quantitative grading. Univariate and multivariate analyses identified predictors of POPF and CR-POPF.

**Results:**

POPF and CR-POPF occurred in 42.5 % and 22.7 % of patients, respectively. Absence of fibrosis and CLII were independently associated with increased risk of POPF (OR 7.51 and 4.30; *p* < 0.0001) and CR-POPF (OR 4.43 and 3.40; *p* = 0.0003 and *p* = 0.0099). Combined absence of both further elevated risk (OR 5.20 for POPF; OR 4.83 for CR-POPF). In multivariate analysis, absence of fibrosis and CLII and main pancreatic duct <3 mm remained independent predictors.

**Conclusion:**

The absence of fibrosis and CLII at the PRM strongly predicts POPF and CR-POPF. Their intraoperative evaluation via frozen sections is feasible and may support tailored surgical strategies, especially in minimally invasive PD.

## Graphical abstract

**Figure:**
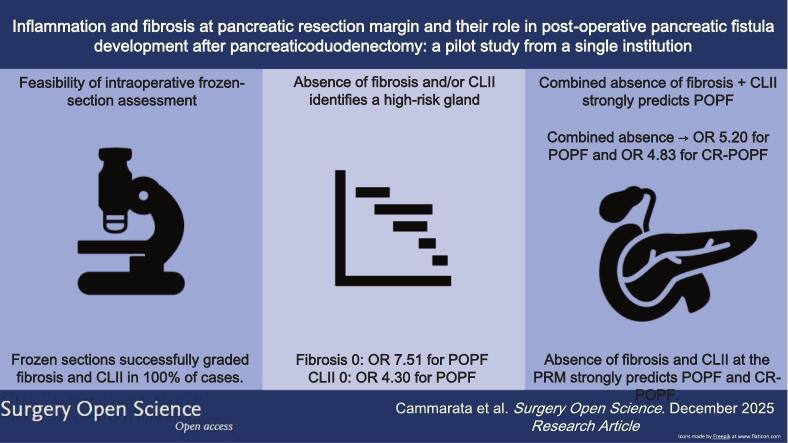

## Introduction

Pancreaticoduodenectomy (PD) is the gold standard for resectable periampullary tumors but remains a technically demanding operation, with complication rates nearing 50 % even in high-volume centers [1]. Despite this, postoperative mortality has dropped below 5 % in specialized units. Among complications, postoperative pancreatic fistula (POPF) is both frequent and feared, affecting about 30 % of patients [2]. It is associated with longer hospital stays, higher costs, and life-threatening events such as bleeding and sepsis [3]. According to the International Study Group of Pancreatic Surgery (ISGPS), POPF is diagnosed when pancreatic amylase levels in drain fluid exceed three times the serum upper limit of normal. It is classified as Grade A (biochemical leak), which is clinically irrelevant, or Grades B and C (clinically relevant POPF, CR-POPF), which worsen outcomes. Mortality for CR-POPF can reach 18.2 % [4,5]. Given the challenge of early prediction, recent strategies emphasize prevention and intraoperative risk stratification [6]. Key predictive factors include main pancreatic duct (MPD) diameter and gland texture, though both rely on subjective intraoperative evaluation. The distinction between soft and firm pancreas is particularly difficult in minimally invasive surgery, where tactile feedback is lacking [2,6,7]. A firm gland often correlates with fibrosis, which may protect against POPF. Separately, chronic inflammatory infiltrates have been suggested as markers of tissue healing, particularly in gastrointestinal anastomoses [8]. However, little is known about local inflammation at the pancreatic resection margin and its role in POPF development. This study aims to identify objective, histological predictors of POPF. Intraoperative frozen sections (IFS) of the pancreatic resection margin (PRM), routinely performed to confirm oncologic radicality, were analyzed. The degree of parenchymal fibrosis and presence of chronic lymphomononuclear inflammatory infiltrate (CLII) were assessed and correlated with the incidence of POPF and CR-POPF in patients undergoing PD.

## Materials and methods

Data from patients undergoing open pancreaticoduodenectomy (PD), performed by two experience surgeons at the General Surgery Division of Fondazione Policlinico Universitario Campus Bio-Medico (Rome) between December 2014 and November 2022 were retrospectively analyzed from a prospectively maintained database. Ethical approval was obtained from the Università Campus Bio-Medico Ethics Committee.

Inclusion criteria were: age > 18 and PD for pancreatic head neoplasms. Exclusion criteria included absence of frozen section analysis of the pancreatic resection margin (PRM), clinical infections at surgery (defined as the presence of an active systemic or local infectious process documented by clinical signs such as fever >38 °C and/or laboratory evidence of infection such as leukocytosis or elevated C reactive protein and/or radiological or microbiological confirmation of conditions including cholangitis, intra-abdominal abscess, pneumonia or urinary tract infection)autoimmune or hematological disorders, or receipt of neoadjuvant treatments. Patients were categorized into two groups: POPF-no vs. POPF-yes, and CR-POPF-no vs. CR-POPF-yes.

All patients underwent preoperative imaging for staging and evaluation of vascular anomalies [9]. When necessary, vascular or multiorgan resections were performed. Pancreaticojejunostomy was performed with a duct-to-mucosa technique. Two abdominal drains were placed: one behind the hepaticojejunal and one behind the pancreatic anastomosis. The diameter of the main pancreatic duct was measured intraoperatively at the transection margin using a sterile ruler, and the value was recorded in millimeters in the operative report. According to literature based cut offs, a threshold of 3 mm was used to stratify patients into high and low risk categories [6]. Pancreatic texture was intraoperatively assessed as soft or firm [6].

Antibiotic prophylaxis was administered by guidelines [11]. Drain amylase levels were measured daily for the first five postoperative days [11]. No specific pharmacological prophylaxis for POPF prevention was used during the study period. Postoperative complications were classified using Clavien-Dindo and ISGPS definitions [5,12].

**Fig. 1:**
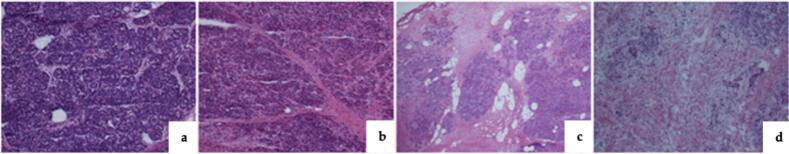
Histopathological classification system of pancreatic fibrosis level (from right to left). (a) Grade 0 = no fibrosis, with normal pancreatic parenchyma; (b) Grade I = periductal fibrosis involving less than 10 % of the pancreatic parenchyma, with preserved lobular architecture; (c) Grade II = periductal and/or diffuse fibrosis affecting 10–90 % of the parenchyma, with varying amounts of fibrotic septa surrounding groups of acini; (d) Grade III = diffuse fibrosis involving more than 90 % of the parenchyma, with only scattered residual acini.

**Fig. 2:**
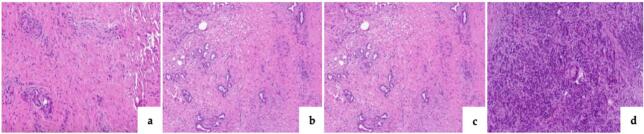
Histopathological classification system of CLII level in the pancreatic parenchyma (from right to left). (a) Grade 0 = no inflammatory infiltrate detected; (b) Grade I = lymphomononuclear infiltrate affecting less than 10 % of the pancreatic parenchyma; (c) Grade II = infiltrate present in 10–70 % of the parenchyma; and (d) Grade III = infiltrate affecting more than 70 % of the pancreatic parenchyma.

H&E-stained slides from PRM intraoperative frozen sections (IFS) were reviewed (Fig. 1, Fig. 2). Fibrosis was graded from 0 to III based on parenchymal involvement [13,14]. Chronic lymphomononuclear inflammatory infiltrate (CLII) was evaluated semi-quantitatively (Grade 0–III) by two senior pathologists (T.C., R.S.).

Statistical analysis was performed using STATA 16. Continuous variables were compared with Student's *t*-test or Mann-Whitney *U* test; categorical variables with Chi-square or Fisher's exact test. ROC curve analysis determined optimal cut-offs. Univariate and multivariate logistic regression identified independent predictors of POPF and CR-POPF. A *p*-value ≤0.05 was considered significant.

## Results

### Demographic data

A total of 360 patients underwent PD during the study period. Among them, 141 met the inclusion criteria and were enrolled in the analysis. The remaining patients were excluded due to the unavailability of frozen section analysis of the pancreatic resection margin. POPF occurred in 60 patients (42.5 %), while CR-POPF was observed in 32 patients (22.7 %). Table 1 summarizes the main demographic and preoperative characteristics of the study population. There were no statistically significant differences between patients who developed POPF and those who did not in terms of age, sex, BMI, ASA score, alcohol consumption, smoking status, or receipt of neoadjuvant therapy. Similarly, no significant differences were found in surgical parameters such as the type of procedure performed, operative time, or intraoperative blood loss. However, a diagnosis of cholangiocarcinoma was significantly associated with a higher incidence of POPF (*p* = 0.0350) in the POPF vs. no POPF group. No other tumor types showed statistically significant associations with POPF or CR-POPF.

**Table 1:** Univariate analysis and relationship of demographics data with POPF and CR-POPF.

Variable	Total, (%)	POPF-no, (%)	POPF-yes, (%)	P-value	CR-POPF-no, (%)	CR-POPF-yes, (%)	P-value
Total, n (%)	141 (100)	81 (57.4)	60 (42.5)	–	109 (77.3)	32 (22.7)	–
Age, y, median (IQR)	72 (65–78)	72 (65–78)	71.5 (64–79)	0.6601	72 (65–78)	71 (63–80)	0.765
Sex, n (%)							
Female	63 (44.6)	36 (44.4)	27 (45)	0.9477	49 (44.9)	14 (43.7)	0.9041
Male	78 (55.3)	45 (55.5)	33 (55)		60 (55)	18 (56.2)	
ASA, n (%)				0.5090			0.0441⁎
I	1 (1.23)	1 (1.66)	0 (0)		1 (0.91)	1 (3.1)	
II	39 (48)	28 (46.6)	11 (18.3)		53 (48.6)	14 (43.7)	
III	37 (45.6)	24 (40)	13 (21.6)		50 (45.8)	11 (34.3)	
IV	4 (4.9)	7 (11.6)	0 (0)		5 (4.5)	6 (18.7)	
BMI, kg/m^2^, median (IQR)	28 (25–31)	28 (25–31)	28.5 (25–32)	0.6456	28 (25–31)	28.5 (25–32)	0.2353
Alcoholic habit, n (%)	3 (2.1)	1 (1.2)	2 (3.3)	0.3932	1 (0.9)	2 (6.25)	0.6568
Smoking habit, n (%)	43 (30.4)	23 (28.3)	20 (33.3)	0.5289	31 (28.4)	12 (37.5)	0.3277
DM, n (%)	31 (21.9)	20 (24.6)	11 (18.3)	0.3674	25 (22.9)	6 (18.7)	0.6152
Hypertension, n (%)	70 (49.6)	41 (50.6)	29 (48.3)	0.0766	54 (49.5)	16 (50)	0.9636
Neoadjuvant treatment, n (%)	10 (7.0)	7 (8.6)	3 (5)	0.4049	10 (9.1)	0 (0)	0.0755
Type of disease, n (%)							
PDAC	110 (78.0)	68 (83.9)	42 (70)	0.0480⁎	88 (80.7)	22 (68.7)	0.1501
AC	3 (2.1)	1 (1.2)	2 (3.3)	0.3932	2 (1.83)	1 (3.1)	0.6566
CCA	11 (7.8)	3 (3.7)	8 (13.3)	0.0350⁎	5 (4.5)	6 (18.7)	0.0086⁎
NET	7 (4.9)	2 (2.4)	5 (8.3)	0.1130	5 (4.5)	2 (6.2)	0.7034
IPMN	6 (4.2)	4 (4.9)	2 (3.3)	0.6406	5 (4.5)	1 (3.1)	0.7186
MCN	3 (2.1)	3 (3.7)	0 (0)	0.1319	3 (2.7)	0 (0)	0.3428
Clear cells	1 (0.7)	0 (0)	1 (1.6)	0.2436	1 (0.9)	0 (0)	0.5866
Intraoperative bleeding, mL, median (IQR)	300 (200–450)	300 (200–450)	300 (200–450)	0.3233	300 (200–450)	300 (200–450)	0.4021
Operative time, min, median (IQR)	375 (330–430)	371 (330–430)	376.5 (330–430)	0.6060	364 (330–430)	382.5 (330–430)	0.6501
DGE, n (%)	50 (35.4)	21 (25.9)	29 (48.3)	0.0060⁎	28 (25.6)	22 (68.7)	<0.0001⁎
PPH, n (%)	24 (17.0)	4 (4.9)	20 (33.3)	<0.0001⁎	8 (7.3)	15 (46.8)	<0.0001⁎
90-day mortality, n (%)	17 (12.0)	6 (7.4)	11 (18.3)	0.0488⁎	11 (10)	6 (18.7)	0.1860
Type of surgery, n (%)				0.3383			0.7583
Whipple	41 (29)	21 (25.9)	20 (33.3)		31 (28.4)	10 (31.2)	
Traverso	100 (71)	60 (74)	40 (66.6)		78 (71.5)	22 (68.7)	

ASA, American Society of Anesthesiologists classification; BMI, body mass index; DM, diabetes mellitus; PDAC, pancreatic ductal adenocarcinoma; AC, ampullary carcinoma; CCA, cholangiocarcinoma; NET, neuroendocrin tumor; IPMN, intraductal papillary mucinous neoplasm; MCN, mucinous cystic neoplasm; DGE, delayed gastric empty; PPH, post-pancreatectomy hemorrhage.

⁎ <0.05.

### Correlation of main pancreatic duct diameter, pancreatic texture and lymphomononuclear inflammatory infiltrate with POPF

The diameter of the MPD was significantly associated with both POPF and CR-POPF: patients with a duct <3 mm had a markedly higher risk of developing POPF (*p* = 0.0001) and CR-POPF (*p* = 0.0031). Similarly, the presence of fibrosis at the pancreatic resection margin was significantly protective against both complications (*p* < 0.0001 for both comparisons).

When these two variables were combined according to the updated ISGPS classification, a significant difference was observed across all categories for both biochemical leak and CR-POPF (*p* < 0.0001 and *p* = 0.0206, respectively), as shown in Table 2.

**Table 2:** Univariate analysis and relationship of risk variables with POPF and CR-POPF.

Variable	POPF-no n (%)	POPF-yes n (%)	P-value	CR-POPF-no n (%)	CR-POPF-yes n (%)	P-value
**MPD diameter**						
<3 mm	22 (27.2)	37 (61.7)	0.0001⁎	38 (34.9)	21 (65.6)	0.00031⁎
>3 mm	59 (72.8)	23 (38.3)		71 (65.1)	11 (34.4)	
**Pancreatic texture (evaluation by surgeon)**						
Soft	41 (50.6)	41 (68.3)	0.0350⁎	63 (57.8)	19 (59.4)	0.8737
Hard	40 (49.4)	19 (31.7)		46 (42.2)	13 (40.6)	
**New ISGPS classification**						
A (hard + ≥3 mm)	27 (33.3)	8 (13.3)	0.0005⁎	30 (27.5)	5 (15.6)	0.0206⁎
B (hard + <3 mm)	13 (16.0)	11 (18.3)		16 (14.7)	8 (25)	
C (soft + ≥3 mm)	32 (39.5)	15 (25)		41 (37.6)	6 (18.7)	
D (soft + <3 mm)	9 (11.1)	26 (43.3)		22 (20.2)	13 (40.6)	
**Pancreatic fibrosis**						
Grade 0	12 (14.8)	34 (56.7)	<0.0001⁎	27 (24.8)	19 (59.4)	0.0062⁎
Grade I	18 (22.2)	17 (28.3)		27 (24.8)	8 (25)	
Grade II	17 (21.0)	8 (13.3)		21 (19.3)	4 (12.5)	
Grade III	34 (42.0)	1 (1.7)		34 (31.1)	1 (3.1)	
**CLII**						
Grade 0	40 (49.4)	48 (80)	0.0049⁎	62 (56.9)	26 (81.2)	0.0934
Grade I	33 (40.7)	11 (18.3)		39 (35.8)	5 (15.6)	
Grade II	7 (8.6)	1 (1.7)		7 (6.4)	1 (3.1)	
Grade III	1 (1.2)	0 (0)		1 (0.9)	0(0)	

MPD, main pancreatic duct; CLII, chronic lymphomononuclear inflammatory infiltrate; POPF, post-operative pancreatic fistula; CR-POPF, clinically relevant POPF.

⁎ Statistically significant.

In the overall cohort, a main pancreatic duct diameter < 3 mm was found in 59 patients (41.8 %), whereas 82 patients (58.2 %) had a duct ≥3 mm. Pancreatic texture was soft in 82 patients (58.2 %) and hard in 59 (41.8 %). According to the updated ISGPS classification, class A was observed in 35 patients (24.8 %), class B in 24 (17.0 %), class C in 47 (33.3 %) and class D in 35 (24.8 %).

Histopathological evaluation of the pancreatic resection margin demonstrated fibrosis grade 0 in 46 patients (32.6 %), grade I in 35 (24.8 %), grade II in 25 (17.7 %) and grade III in 35 (24.8 %). CLII was absent (grade 0) in 88 patients (62.4 %), while grade I, II and III infiltrate were found in 44 (31.2 %), 8 (5.7 %) and 1 (0.7 %) patients, respectively.

Histopathological analysis of IFS from the pancreatic resection margin demonstrated a significant correlation between the degree of pancreatic parenchymal fibrosis and the presence of lymphomononuclear inflammatory infiltrate, graded into four levels as explained in material section, with the risk of developing POPF and CR-POPF, both when evaluated individually and in combination. As shown in Table 3, the absence of pancreatic fibrosis was associated with a significant higher risk of POPF (OR 7.51; *p* < 0.0001) and CR-POPF (OR 4.43; *p* < 0.0001). Similarly, the absence of lymphomononuclear inflammatory infiltrate (LII) was linked to a higher risk of POPF (OR 4.30; p < 0.0001) and CR-POPF (OR 3.40; *p* = 0.0099). When neither inflammatory infiltrate nor pancreatic fibrosis were present, the risk of POPF and CR-POPF further increased [(OR 5.20; p < 0.0001), (OR 4.83; *p* = 0.020), respectively].

**Table 3:** Correlation between fibrosis and CLII with POPF and CR-POPF.

Variable	POPF-no vs. POPF-yes			CR-POPF-no vs. CR-POPF-yes		
	OR	95 % CI	P-value	OR	95 % CI	P-value
Grade 0 fibrosis	7.51	3.08–18.3	<0.0001⁎	4.43	1.85–10.6	0.0003⁎
Grade 0 CLII	4.30	1.90–9.73	0.0001⁎	3.40	1.26–9.19	0.0099⁎
Grade 0 fibrosis and CLII	5.20	2.23–12.1	<0.0001⁎	4.83	1.27–18.3	0.0200⁎

⁎ <0.05.

The degree of pancreatic fibrosis determined histopathologically has shown a significant correlation with the surgical assessment of pancreatic texture (p < 0.0001), as depicted in Table 4 (Table 4).

**Table 4:** Correlation of surgical and pathological evaluation of pancreatic texture.

Histopathological evaluation	Surgeon evaluation		
Pancreatic fibrosis	Pancreatic texture		P-value
	Hard (59)	Soft (82)	
Grade 0	10 (16.9)	36 (43.9)	<0.0001⁎
Grade I	9 (15.3)	26 (31.7)	
Grade II	17 (28.8)	8 (9.8)	
Grade III	23 (39.0)	12 (14.6)	

⁎ Statistically significant.

### Multivariate analysis

The multivariate analysis identified a main pancreatic duct diameter of less than 3 mm and the combination of fibrosis and inflammation as independent risk factors for both POPF [OR 4.22 (1.80–9.88), p = 0.001] and CR-POPF [OR 3.1 (1.19–8.07), *p* = 0.020]. Additionally, the combination of fibrosis and inflammation was also a significant predictor for POPF [OR 5.44 (2.11–14.00)] and CR-POPF [OR 4.83 (1.27–18.3), *p* = 0.020]. The results of the multivariate analysis are detailed in Table 5.

**Table 5:** Multivariate logistic regression identifying independent predictors of POPF and CR-POPF.

Variable	No POPF vs. POPF			No CR-POPF vs. CR-POPF		
	OR	95 % CI	P-value	OR	95 % CI	P-value
Age, y	1.02	0.97–1.07	0.329	0.99	0.94–1.04	0.754
Sex, male vs. female	1.35	0.59–3.04	0.467	1.48	0.58–3.75	0.404
BMI > 30 kg/m^2^	2.61	0.71–9.59	0.148	3.12	0.59–16.4	0.178
NAT, yes vs. no	1.35	0.52–3.50	0.536	1		
Operative time	1.00	0.99–1.00	0.377	0.99	0.99–1.00	0.453
Blood loss, >300 mL	0.99	0.99–1.00	0.415	1.00	0.99–1.00	0.512
MPD < 3 mm	4.22	1.80–9.88	0.0001⁎	3.1	1.19–8.07	0.020⁎
No fibrosis and CLII	5.44	2.11–14.00	0.0001⁎	4.83	1.27–18.3	0.020⁎

⁎ <0.05.

## Discussion

Despite advances in perioperative care and the increased adoption of minimally invasive surgery and neoadjuvant chemotherapy, CR-POPF remains a prevalent and impactful complication after pancreaticoduodenectomy. A recent large-scale analysis has shown a rising incidence of CR-POPF, from 13.3 % to 15.7 % over a five-year period, underscoring the limited effectiveness of current strategies in preventing this outcome. [15]

Historically, risk stratification relied on postoperative findings or intraoperative scoring systems, mainly to guide early detection and postoperative surveillance. However, the growing clinical burden of POPF and its consequences on the administration of adjuvant therapy have shifted attention toward the development of intraoperative predictive tools that allow for immediate, personalized decision-making. [16] This paradigm shift, from reactive to proactive, demands objective, reproducible markers that can be integrated into surgical planning and guide intraoperative strategies such as drain placement, pharmacological prophylaxis, or even total pancreatectomy in selected high-risk cases [[17], [18], [19]].

Among the numerous risk factors for POPF, the histological features of the remnant pancreatic parenchyma are the most strongly correlated. In particular, the presence of fibrosis, by increasing tissue firmness, has been shown to enhance suture stability, hence lowering the risk of pancreatico-jejunal anastomotic leakage [20]. However, the assessment of these features remains highly variable, primarily due to its reliance on the surgeon's intraoperative judgment. This variability is further exacerbated by the lack of tactile feedback in minimally invasive procedures and the absence of standardized definitions, making the evaluation of pancreatic texture inherently subjective [21].

Our data supports the hypothesis that histopathological features assessed intraoperatively at the pancreatic resection margin may provide objective markers for POPF risk stratification. Multivariate analysis identified the absence of fibrosis as strongly associated with the development of POPF (OR 7.51, 95 % CI 3.08–18.3; *p* < 0.0001) and CR-POPF (OR 4.43, 95 % CI 1.85–10.6; *p* = 0.0003). Similarly, a main pancreatic duct diameter below 3 mm was independently associated with increased risk of POPF (OR 4.22, 95 % CI 1.80–9.88; *p* = 0.001) and CR-POPF (OR 3.10, 95 % CI 1.19–8.07; *p* = 0.020). These findings are consistent with existing literature and reinforce the clinical relevance of these parameters in perioperative risk assessment [13].

Although the histological grading system used in this study is semi-quantitative, it is based on well-established morphological criteria applied to routine hematoxylin and eosin (H&E)-stained frozen sections. This methodological simplicity allows for practical intraoperative implementation and, when performed by trained pathologists, may offer a reproducible alternative to the surgeon's subjective assessment, especially valuable in minimally invasive procedures where tactile feedback is lacking.

Simultaneously, the role of inflammation in the development of surgical complications has been widely investigated. Numerous studies in pancreatic surgery have highlighted the significant impact of systemic inflammation on postoperative outcomes, particularly in predicting the occurrence of CR-POPF [10].

In addition, it has been suggested that acute inflammation of the residual pancreatic parenchyma following resection may contribute to the development of POPF. In this regard, the ISGPS has recently introduced the concept of post-pancreatectomy acute pancreatitis (PPAP), recognizing it as a distinct clinical entity potentially linked to pancreatic fistula formation [22].

While systemic and acute inflammation have been extensively studied, the role of chronic inflammation in this setting remains poorly defined. Nonetheless, it is well established that chronic inflammatory infiltrates play a critical role in wound healing. Lymphocytes are central in regulating the balance between inflammation and immune tolerance. This balance is essential to ensure that the inflammatory response initiated to control infection and trigger tissue repair does not become excessive, thereby preventing collateral tissue damage. In addition, lymphocytes contribute to the modulation of the immune response by promoting cell proliferation and the formation of new tissue. These mechanisms are crucial for achieving effective and well-regulated wound healing [23].

The presence of a robust chronic inflammatory infiltrate at the pancreatic transection margin could therefore represent an additional surrogate marker of fibrotic pancreatic parenchyma, potentially conferring protection against anastomotic leakage. A well-structured chronic inflammatory response may contribute to a local microenvironment that supports tissue regeneration and improves anastomotic integrity [24].

In our study, the absence of CLII was significantly associated with a higher risk of POPF (OR 4.30, 95 % CI 1.90–9.73; *p* = 0.0001) and CR-POPF (OR 3.40, 95 % CI 1.26–9.19; *p* = 0.0099). Furthermore, when both fibrosis and CLII were absent, the risk increased further (OR 5.20 for POPF, *p* < 0.0001; OR 4.83 for CR-POPF, *p* = 0.020). These findings suggest that CLII, much like fibrosis, may reflect a tissue state conducive to effective healing and lower anastomotic vulnerability. To our knowledge, this is the first study to systematically investigate chronic inflammatory infiltrate as an intraoperative histological marker for POPF risk. While the precise biological mechanisms remain to be clarified, these results open new perspectives on the role of local tissue inflammation in modulating surgical outcomes. If validated in larger series, CLII could be incorporated into standardized intraoperative pathology workflows to support real-time surgical decision-making.

To our knowledge, this is the first study to propose a structured and reproducible method for assessing chronic inflammatory infiltrates in the pancreatic parenchyma using intraoperative frozen sections and no studies have evaluated the sensitivity or specificity of intraoperative frozen section for the assessment of fibrosis or chronic inflammatory infiltrate at the pancreatic transection margin. Our data demonstrate that the absence of CLII in the residual pancreatic tissue is significantly associated with an increased risk of both POPF and CR-POPF. Notably, this association is further amplified in the simultaneous absence of fibrosis, suggesting that the lack of both features defines a particularly high-risk pancreatic remnant. These findings are consistent with established pathophysiological concepts linking chronic inflammatory infiltrates to tissue regeneration and wound healing. In this context, a robust CLII may reflect not only a marker of underlying fibrosis but also an active microenvironment favorable to anastomotic integrity. Conversely, the simultaneous absence of both fibrosis and CLII may identify a biologically and mechanically fragile pancreas more prone to leakage. Further studies are warranted to validate these observations in larger, multicenter cohorts and to explore their integration into intraoperative decision-making algorithms. If confirmed, the combined assessment of fibrosis and CLII could represent a valuable adjunct to existing risk stratification tools, particularly in the setting of minimally invasive pancreatic surgery. [25]. This study presents some limitations that should be acknowledged. Its retrospective and single-center design limits the generalizability of the findings and may introduce selection and institutional biases. Although histological grading was performed using predefined criteria, interobserver variability was not formally assessed, and consistency across institutions remains to be validated. Furthermore, *the study does not include minimally invasive procedures, as all pancreaticoduodenectomies performed during the study period were open*. Future prospective, multicenter studies are warranted to confirm the reproducibility and clinical relevance of this approach in diverse settings especially in minimally invasive one.

## Conclusions

This study introduces several novel elements when contextualized within the current literature. While numerous investigations have examined the role of systemic inflammation in predicting major surgical complications, limited attention has been directed toward the state of local inflammation, particularly within the tissues directly involved in the anastomotic site [26].

The key innovation of our work lies in the development of a reproducible intraoperative tool for assessing the risk of POPF, based on the presence of fibrosis and CLII. This approach relies on the immediate evaluation of frozen sections from the PRM by an experienced pathologist. As demonstrated by our results, this method offers clinically relevant information that can be used to guide intraoperative decisions aimed at minimizing the incidence and severity of CR-POPF. In line with recent literature, identifying a high-risk pancreas, characterized by the absence of both fibrosis and CLII, could support the adoption of more protective strategies, such as drain placement or, in selected cases, total pancreatectomy. Conversely, a parenchyma rich in fibrosis and inflammation may justify a more conservative approach, including the selective omission of surgical drains [[27], [28], [29]].

As pancreatic surgery increasingly embraces minimally invasive techniques, the surgeon's ability to assess gland texture intraoperatively becomes more limited due to the absence of tactile feedback. In this setting, the development of objective, pathology-based tools for POPF risk assessment becomes particularly valuable to preserve the benefits of laparoscopic and robotic approaches [30].

We believe that integrating such histological markers into intraoperative workflows may assist surgeons in tailoring the operative strategy to individual patient risk. Nonetheless, this study has several limitations. The relatively small sample size and the retrospective, single-center design may limit the generalizability of the findings and introduce potential biases. Further prospective studies, particularly in minimally invasive settings, are warranted to validate these observations and to explore their integration into existing risk models and perioperative management protocols.

## Limitations

This study has several limitations. First, its retrospective and single-centre design may limit the generalizability of the findings. Second, although histological grading of fibrosis and chronic lymphomononuclear inflammatory infiltrate (CLII) was performed by two senior pathologists using predefined criteria, interobserver variability was not formally assessed. Third, the sample size, although representative of our institutional practice, remains relatively limited. Finally, all pancreaticoduodenectomies in this cohort were performed using an open approach, preventing any meaningful analysis of the impact of minimally invasive techniques.

## CRediT authorship contribution statement

**Roberto Cammarata:** Resources, Project administration, Methodology, Investigation, Formal analysis, Data curation, Conceptualization. **Vincenzo La Vaccara:** Formal analysis. **Alberto Catamerò:** Formal analysis, Data curation, Conceptualization. **Chiara Taffon:** Writing – original draft, Formal analysis, Conceptualization. **Gianluca Costa:** Data curation, Conceptualization. **Laura Olivieri:** Formal analysis, Data curation. **Roberto Coppola:** Supervision. **Damiano Caputo:** Supervision.

## Informed consent statement

Patient consent was waived due to the retrospective design of the study and considering that data are de-identified.

## Institutional review board statement

The study was conducted according to the guidelines of the Declaration of Helsinki and approved by the Ethics Committee of Università Campus Bio-Medico di Roma (protocol code 105.20 OSS.NOT; 2/12/2020).

## Funding

This research received no external funding.

## Declaration of competing interest

The authors declare that they have no known competing financial interests or personal relationships that could have appeared to influence the work reported in this paper.

## Data Availability

The data presented in this study are available on request from the corresponding author.
